# Microbiome and virome on indoor surfaces of an Antarctic research ship

**DOI:** 10.1590/0074-02760230084

**Published:** 2023-09-04

**Authors:** Tatiana Prado, Maithê Gaspar Pontes Magalhães, Daniel Andrade Moreira, Martha Lima Brandão, Tulio Machado Fumian, Fernando Cesar Ferreira, Marcia Chame, Luciana Leomil, Wim Maurits Sylvain Degrave, José Paulo Gagliardi Leite, Marize Pereira Miagostovich

**Affiliations:** 1 Fundação Oswaldo Cruz-Fiocruz, Laboratório de Vírus Respiratórios, Exantemáticos, Enterovírus e Emergências Virais, Rio de Janeiro, RJ, Brasil; 2 Fundação Oswaldo Cruz-Fiocruz, Laboratório de Genômica Aplicada e BioInovações, Rio de Janeiro, RJ, Brasil; 3 Fundação Oswaldo Cruz-Fiocruz, Projeto FioAntar/VPPIS, Rio de Janeiro, RJ, Brasil; 4 Fundação Oswaldo Cruz-Fiocruz, Laboratório de Virologia Comparada e Ambiental, Rio de Janeiro, RJ, Brasil; 5 Fundação Oswaldo Cruz-Fiocruz, Plataforma Institucional para Biodiversidade e Saúde Animal, Rio de Janeiro, RJ, Brasil; 6 Serviço Nacional de Aprendizagem Industrial, Centro Tecnológico para Indústria Química e Têxtil, Biotecnologia, Parque Tecnológico da Universidade Federal do Rio de Janeiro, Rio de Janeiro, RJ, Brasil

**Keywords:** Antarctica, metagenomics, microbiome, naval indoor surfaces, virome

## Abstract

**BACKGROUND:**

Few studies have focused on microbial diversity in indoor environments of ships, as well as the role of the microbiome and its ecological interconnections. In this study, we investigated the microbiome and virome present on the internal surfaces of a polar ship in different stages (beginning, during, and at the end) of the Brazilian Antarctic expedition in order to evaluate abundance of microorganisms in different periods.

**OBJECTIVES AND METHODS:**

We used shotgun metagenomic analysis on pooled samples from sampling surfaces in the ship’s interior to track the microbial diversity.

**FINDINGS:**

Considering the total fraction of the microbiome, the relative abundance of bacteria, eukaryotes, viruses, and archaea was 83.7%, 16.2%, 0.04%, and 0.002%, respectively. *Proteobacteria* was the most abundant bacterial phyla, followed by *Firmicutes*, *Actinobacteria*, and *Bacteroidetes*. Concerning the virome, the greatest richness of viral species was identified during the middle of the trip, including ten viral families after de novo assembly: *Autographiviridae*, *Chrysoviridae*, *Genomoviridae*, *Herelleviridae*, *Myoviridae*, *Partitiviridae*, *Podoviridae*, *Potyviridae*, *Siphoviridae*, and *Virgaviridae*.

**MAIN CONCLUSIONS:**

This study contributed to the knowledge of microbial diversity in naval transportation facilities, and variations in the abundance of microorganisms probably occurred due to factors such as the number of passengers and activities on the ship.

Ships are semi-closed and densely populated environments of close living and sleeping quarters, shared water, meals, and ventilation and sewage systems.^1^ Because of these specificities, ships are considered potential sources of disease outbreaks, and promoters of transmission of pathogens already present or introduced on board. Therefore, the spread of vectors and microorganisms from crew and passengers to land-based populations and vice versa should be considered.^2^ ^,^ ^3^ ^,^ ^4^ ^,^ ^5^ This last assertion is particularly relevant considering expeditions to the Antarctic continent, due to the risk of introducing and spreading exotic or autochthonous species in a relatively unexplored and untouched continent.

Microbial infections are probably the most common acquired diseases indoors, mainly in poorly ventilated environments.^6^ ^,^ ^7^ ^,^ ^8^ Pathogens such as norovirus, influenza virus, *Legionella* spp., *Salmonella* spp., *E. coli*, *Vibrio* spp., *Mycoplasma pneumoniae*, as well as vaccine-preventable diseases such as measles, rubella, and varicella are well documented in cruise ships and military vessels.^2^ ^,^ ^3^ ^,^ ^9^ ^,^ ^10^ ^,^ ^11^ ^,^ ^12^ More recently, the transmission of severe acute respiratory syndrome coronavirus-2 (SARS-CoV-2) in cruise and military ships, among other transportation facilities, has been well documented^4^ ^,^ ^5^ ^,^ ^13^ with implications not only for the health of the crew but also contributing to the spread of SARS-CoV-2 among different coastal locations and continents.^13^

In the context of a polar expedition, the researchers also collect samples from the soil, sea, lakes, ice, and animal excrement. They can transport microorganisms in the polar ecosystem to the ship through contact with these matrices. The primary issue is due to the dispersal and adaptive capacities of some organisms, mainly pathogens, to the other continents and eventually introducing ecological and public health risks.

Microorganisms are deposited in the environment when excreta or other secretions (such as mucus, saliva, urine, and faeces) containing high concentrations or viral titres are released from an infected individual.^14^ ^,^ ^15^ ^,^ ^16^ For instance, faeces can contain up to 10^12^ viral particles per gram and vomit up to 10^7^ per millilitre, so the potential cross-contamination from hands to surfaces is considerable.^1^ ^,^ ^14^ Moreover, other significant sources of indoor microorganisms may be human oral and respiratory fluid emitted via coughing, sneezing, talking, and breathing or the direct shedding of skin-associated microbiota.^6^ ^,^ ^7^ ^,^ ^17^ Toilet flushing can also aerosolise significant concentrations of viruses.^1^

Viruses can be transmitted through person-to-person contact or waterborne, foodborne, airborne, and vector-borne. In addition, the high stability of viruses or other microorganisms on surfaces or fomites emphasises the possible role of surfaces in the transmission route, mainly through contact.^3^ ^,^ ^14^ ^,^ ^16^ ^,^ ^18^ ^,^ ^19^ Both enveloped and nonenveloped viruses are readily transferred between fomites and fingerpads, with an estimated transfer rate of ~22%.^16^ Therefore, fomites are essential vehicles for the spread of pathogens and associated diseases.^14^ ^,^ ^20^ ^,^ ^21^ ^,^ ^22^

In addition to pathogens that may be present on surfaces, recent studies have demonstrated that indoor surfaces of transportation facilities are microbial reservoirs from multiple sources. Human presence and nearby surroundings contribute to the characteristics of the microbiome.^6^ ^,^ ^7^ ^,^ ^8^ ^,^ ^23^ ^,^ ^24^ ^,^ ^25^ Some factors can influence the microbial composition on indoor surfaces including material types, moisture rates, temperature, cleaning practices, human occupancy, and occupant activities.^6^ ^,^ ^23^ ^,^ ^24^ ^,^ ^25^ ^,^ ^26^ However, more information is needed about microbiome variations on indoor surfaces in transportation facilities under different physical-chemical conditions, types of materials, transport routes, number of passengers, distinct activities, and geographic location.^6^ ^,^ ^8^

In this study, we analysed the microbiome and virome, including target viruses with RNA (ribonucleic acid) genome, from swab samples collected on indoor environmental surfaces of a Brazilian Navy Polar vessel (Almirante Maximiano - H41). For this, we used a metagenomic approach to track the microbial diversity during different stages of an expedition to Antarctica, considering other activities and the number of passengers on the ship. Swab samples collected on several surfaces during various expedition stages were pooled to assess the abundance of microorganisms present at each stage and whether autochthonous or allochthones environmental species could be carried between different continents.

## MATERIALS AND METHODS

**Fig. 1:**
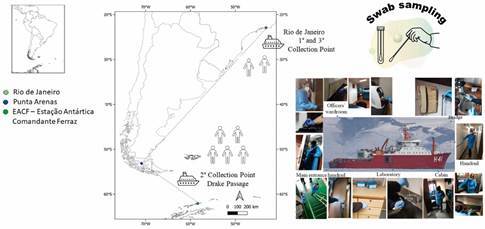
swab samples obtained from different surfaces during three stages of a Brazilian Antarctic expedition (Brazilian Navy Polar vessel - Almirante Maximiano H41 - October 2019 to April 2020).

*Sampling* - Ninety-one swab samples from fomites and surfaces were obtained from different indoor environments (Fig. 1, Table I) according to a protocol by Ganime et al.,^19^ with minor modifications. Briefly, samples were obtained by swabbing at least 50% of a selected surface area with rayon swabs dipped and stored in 2.0 mL Dulbecco′s Phosphate Buffered Saline (PBS, 1.5X), pH 7.2. Swab samples were collected in the same places at three different periods during the Brazilian Antarctic expedition in 2019/2020 (Fig. 1). The first collection took place on October 7th, 2019, in Rio de Janeiro port, before the departure of the ship for the expedition (n = 34 samples); the second on February 3rd, 2020, while crossing the Drake Passage and anchoring in the port of Punta Arenas, Chile (n = 32 samples); and the third on April 5th, 2020, when the ship arrived in the port of Rio de Janeiro, Brazil, at the end of the expedition (n = 25 samples) (Fig. 1, Table I). These three periods were chosen to assess the microbial communities before the ship’s departure, during the expedition, when a more significant number of people were on board, with frequent changes of part of the passengers, and at the end of the expedition to assess the potential microbial spread in the indoor environment. At the beginning and end of the trip, when the ship is moored, there is no crew on board, only a few navy officers responsible for the ship’s maintenance. During the expedition, about 110 crew members were on board (https://www.marinha.mil.br/navio-polar-almirante-maximiano), including researchers and navy personnel. Before the voyage, the ship’s surfaces are decontaminated to receive the crew. At the end of the expedition, the vessel underwent a surface decontamination process before docking in Rio de Janeiro, Brazil. Such actions were intensified after the decree made by the World Health Organization (WHO) in March 2020 of the coronavirus disease 19 (COVID-19) pandemic.

**TABLE I:** Sampling and swab collection locations

Sampling site	Swab collection (material type)
Laboratory	Faucets (metal), cabinet and freezer knobs (metal), computer / keyboard and mouse (plastic)
Cabins (n = 2)	Intercom (plastic), Beds (wooden sides), switches (plastic)
Cabin bathrooms (n = 2)	Faucet (metal), door handles (metal), toilet handrail (metal), flush button (metal)
Officers’ wardroom	Access handrail, deck handrail, TV controls (plastic), laptop keyboards (plastic), chairs (wood), intercom (plastic), air conditioning (remote control and refrigeration equipment) (plastic and metal), drinking fountain (plastic), cafeteria and milk jug (plastic), bell (rope) (metal)
Toilet of officers’ wardroom	Door handles (metal), flush button (metal), toilet handrail (metal), faucets (metal)
Bridge Gangway	Handrail (iron)

Surface samples were collected in places of greater crew circulation, such as cabins, restrooms, and handrails, where people touched more frequently (Fig. 1, Table I).

After swab collection, samples were processed immediately (stages 1 and 3) or kept under refrigeration at 4ºC during the trip (stage 2), until processed at the Laboratory of Comparative and Environmental Virology at Instituto Oswaldo Cruz (FIOCRUZ/RJ). In all periods, one swab sample was collected per location indicated in Table I, except for some examples collected in duplicate at the beginning of the expedition (October 7th, 2019) in specific fomites, such as faucets, cabinets, and freezer knobs. However, in subsequent collections, we considered it unnecessary to sample in duplicate. For metagenomics analysis, samples collected in each sampling period were pooled (10 µL from each sample kept in PBS solution) totalling three pools (named pool 1: collection carried out on October 7th, 2019 (n = 34); pool 2: collection carried out on February 3rd, 2020 (n = 32), and pool 3: collection carried out on April 5th, 2020 (n = 25).

*Nucleic acid extraction, reverse transcription, preparation of genomic libraries, and sequencing* - Samples were prepared according to the protocol described by Fernandez-Cassi et al.^27^ Briefly, 150 **μL** of each pooled sample was treated with 160 U of Turbo DNAse (Ambion Cat no. AM1907, Ambion) for 1 h at 37**º**C to remove free DNA (Deoxyribonucleic acid). DNAse was inactivated using the provided inactivation reagent, and the samples were centrifuged at 10,000 ×g for 1.5 min. The treated supernatants were collected, and 140 µL of the DNAse treated samples were extracted using the QIAamp^®^ Viral RNA Mini kit (QIAGEN, CA, USA) in a QIAcube^®^ automated system (QIAGEN) without the addition of RNA carrier. RNA templates were reverse transcribed precisely as Fernandez-Cassi et al.^27^ to identify viruses with RNA genomes. The second cDNA strand construction, and a polymerase chain reaction (PCR) amplification step were performed to obtain sufficient DNA for library preparation.^27^ ^,^ ^28^ PCR products were purified, and concentrated to a volume of 50 µL using the Agencourt AMPure XP PCR purification kit (Beckman Coulter, CA, USA). Negative controls (DNase/RNase free water) were included in all stages of the procedures, and agarose gel electrophoresis was performed to verify DNA amplification, as described by Wang et al.^28^

The purified amplicons were quantified using Qubit 2.0, and DNA libraries were generated using a Nextera XT DNA Preparation Kit (Illumina, San Diego, CA, USA). The size distribution of the libraries was evaluated using a 2100 Bioanalyzer (Agilent, Santa Clara, CA, USA), and DNA High Sensitivity quantification was obtained using a Qubit 4.0 Fluorometer. Paired-end sequencing (2 x 150 bp) was performed using the NextSeq platform (Illumina, San Diego, CA, USA) at SENAI CETIQT’s Facility (SENAI Innovation Institute for Biosynthetics, Technology Centre and Textile Industry, Rio de Janeiro, RJ, Brazil) and PhiX was used as a control for Illumina sequencing runs.

*Bioinformatics and data analysis* - The reads in FASTQ format were generated by the Illumina BaseSpace pipeline (https://basespace.illumina.com). Low-quality sequences were filtered (Phred score < 20), and adapters were removed with trimmomatic v0.39.^29^ The read quality analyses were performed using FastQC v0.11.9^30^ before and after trimming. The software Kraken2 was used for a metagenomic and taxonomic assignment using the standard Kraken2_DB database.^31^ The relative abundance estimates of microorganisms were refined using Bracken software.^32^ Classified microorganisms with less than 10 reads were excluded from the relative abundance analysis.

Reads were *de novo* assembled using metaSPAdes^33^ for paired-end reads. Contigs longer than 150 bp were queried for sequence similarity search using Blastx (parameters: e-value 1e-10 -max_target_seqs 25)^34^ against the NCBI RefSeq database. Subsequently, BASTA (Basic Sequence Taxonomy Annotation) (parameters: -m 1 -l 75 -i 70),^35^ was used to determine the taxonomy annotation of Blastx hits based on a last common ancestor algorithm. The species’ nomenclature and classification were according to the NCBI (National Centre for Biotechnology Information) Taxonomy database standards (https://www.ncbi.nlm.nih.gov/guide/taxonomy/).

Each viral contig (≥ 150 bp) was manually checked through Blastx, and the predicted viral hosts were inferred based on the closest relative sequence (lower e-value and higher score and nucleotide identity) found in the database by protein alignment along with information reported by the International Committee on Taxonomy of Viruses (ICTV - Report on Virus Classification and Taxon Nomenclature) (https://talk.ictvonline.org/ictv-reports/ictv_online_report/).

*Data availability* - Raw reads are publicly available in the Sequence Read Archive (SRA) (NCBI - https://www.ncbi.nlm.nih.gov/sra) individually with accession numbers (SRX15809764 to SRX15809766) under BioProject accession number (PRJNA850925).

## RESULTS

*Microbial diversity* - A total of 19,502,486 paired-end reads were obtained from the sequencing of the three pools of samples. Table II shows the number of reads before and after trimming, low-quality sequences filtering, and the percentage of reads obtained for each biology domain obtained in each metagenomic library after taxonomic classification.

**TABLE II:** Number of raw reads and after quality control, percentage of reads for each domain obtained in each metagenomic library according to different pooled samples

Pool*	No. of raw reads	Number of reads (after quality control)	*Bacteria*	*Archaea*	*Eukarya*	Virus
1	6,357,049	4,937,444	4,411,561(95.5%)	27 (0.0005%)	206,718 (4.4%)	173 (0.003%)
2	3,691,839	2,611,274	71,462 (3.2%)	231 (0.01%)	2,161,503 (96.5%)	6,247 (0.3%)
3	9,453,598	8,101,341	7,898,636 (99.5%)	59 (0.0007%)	34,119 (0.4%)	71 (0.0008%)
Total	19,502,486	15,650,059	12,381,659	317	2,402,340	6,491

*pool 1: beginning of expedition (Stage 1), pool 2: during the expedition (Stage 2), pool 3: end of expedition (Stage 3).

The analysis shows dominance of bacteria in the pooled samples corresponding to the beginning (stage 1) and the end of the expedition (stage 3). In contrast, pool 2, representing samples from the fully crewed ship, the Eukarya domain was dominant (Table II).

Although the fraction of *Archaea* was tiny concerning the percentage observed for the other domains (abundance ≤ 0.01%) (Table II), it was possible to follow the occurrence of *Crenarchaeota*, *Euryarchaeota*, and *Lokarchaeota* phylum in all metagenomic libraries.

**Fig. 2:**
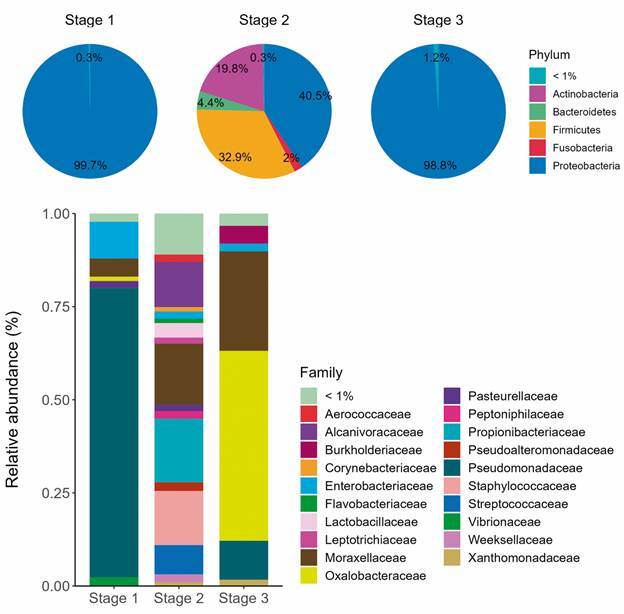
(A) Relative abundance (%) of bacterial phyla present in each pooled sample of indoor surfaces of the ship during different stages of an Antarctic expedition (2019/2020). (Stage 1: before the expedition; Stage 2: during the expedition; Stage 3: end of the expedition). (B) Relative abundance (%) of bacterial families identified in each pooled sample collected on indoor surfaces of a Navy Polar ship (2019/2020).

Fig. 2A shows the relative abundance of bacterial phyla in each pooled sample concerning the total bacterial fraction obtained. *Proteobacteria* was the most abundant phyla in all samples, representing 99.7%, 40.5%, and 98.8% in stages 1, 2, and 3, respectively. After *Proteobacteria* phyla, *Firmicutes*, *Actinobacteria* and *Bacteroidetes* were the following most abundant phyla in steps 1 (0.2, 0.09, 0.01%, respectively), and 2 (32.9, 19.8, 4.4%, respectively), while in the stage 3 *Proteobacteria* were followed by *Bacteroidetes* (0.6%), *Actinobacteria* (0.4%) and *Firmicutes* (0.1%) (Fig. 2A). In stage 2 *Fusobacteria* was the fifth most abundant phyla (2%), while other species represented less than 1% in the metagenomic library (Fig. 2A).

*Pseudomonadaceae* family was most abundant (77.6%) in stage 1 compared with a total number of bacterial reads obtained, followed by *Enterobacteriaceae* (9.8%), *Moraxellaceae* (4.8%), *Vibrionaceae* (2.3%), *Pasteurellaceae* (1.8%), *Oxalobacteraceae* (1.1%) (Fig. 2B). In stage 2, *Propionibacteriaceae* was predominant (17%), followed by *Moraxellaceae* (16.3%), *Staphylococcaceae* (14.5%), *Alcanivoracaceae* (12.1%), *Streptococcaceae* (7.8%), and *Lactobacillaceae* (3.9%) (Fig. 2B). *Oxalobacteraceae* family was predominant in stage 3 (50.9%), followed by *Moraxellaceae* (26.7%), *Pseudomonadaceae* (10%), *Burkholderiaceae* (4.8%), *Enterobacteriaceae* (2%), and *Xanthomonadaceae* (1.6%) (Fig. 2B). Other families presented percentages lower than 1% in the metagenomic libraries.

In stage 1, after the most abundant *Pseudomonas* genera (77.5%), *Samonella* was the second largest (8.0%), followed by *Acinetobacter* (4.6%), *Vibrio* (2.2%), *Actinobacillus* (1.8%), *Massilia* (1.1%) and other ≤ 1%. *Cutibacterium* and *Staphylococcus* were the first and second most abundant bacterial genus (17% and 14.5%, respectively) in stage 2, followed by *Acinetobacter* (12.4%), *Alcanivorax* (12%), *Streptococcus* (7.7%), *Moraxella* (3%), *Pseudoaltermonas* (2.3%), *Aerococcus* (1.7%), *Finegoldia* (1.6%), *Weissella* (1.5%), *Haemophilus* (1.4%), *Chryseobacterium* (1.3%), *Leptotrichia* and *Corinebacterium* (1.2%), *Stenotrophomonas* (1%), and other ≤ 1%. In stage 3, *Massilia* genus (50.7%) was predominant, followed by *Psychrobacter* (19%), *Pseudomonas* (10%), *Acinetobacter* (7.6%), *Burkholderia* (4.6%), *Stenotrophomonas* (1.3%) and other ≤ 1%.

Some bacterial species related to the Antarctic continent were observed in the samples collected during the different stages of the expedition (Table III). Table III presents the results of the taxonomic classification obtained for other bacterial species related to the Antarctic environment and their relative abundance by pooled sample. The relative abundance of bacteria associated with the Antarctic was very low concerning the total quantity found for other species (Table III).

**TABLE III:** Taxonomic classification of bacterial hits related to the Antarctic continent obtained in swab samples collected on indoor surfaces of an Antarctic research ship during different stages of the expedition in 2019/2020

Stages	Species (Reference ID - NCBI)	Number of reads (Relative abundance %)	The probable source of isolation*
1	*Pseudomonas Antarctica* (219572)	29 (≤ 0.00001)	cyanobacterial mat samples that were collected from various water bodies in Antarctica
	*Pseudolysobacter antarcticus* (2511995)	101 (≤ 0.00001)	soil in Fildes Peninsula, Antarctica
	*Granulosicoccus antarcticus* (437505)	373 (0.01)	Antarctic coastal seawater
	*Acidovorax antarcticus* sp. (2743470)	89 (≤ 0.00001)	soil sample of Collins Glacier front, Antarctica
	*Rhodoferax antarcticus* (81479)	44 (≤ 0.00001)	Antarctic microbial mat
2	*Rhodoferax antarcticus* (81479)	108 (≤ 0.00001)	Antarctic microbial mat
	*Acidovorax antarcticus* sp. (2743470)	14 (≤ 0.00001)	soil sample of Collins Glacier front, Antarctica
	*Massilia Antarctica* (2765360)	42 (≤ 0.00001)	freshwater samples collected in a deglaciated part of James Ross Island and Eagle Island, Antarctica (2017-2019)
3	*Acidovorax antarcticus* sp. (2743470)	3,453 (0.04)	soil sample of Collins Glacier front, Antarctica
	*Pseudolysobacter antarcticus* (2511995)	243 (≤ 0.00001)	soil in Fildes Peninsula, Antarctica
	*Granulosicoccus antarcticus* (437505)	211 (≤ 0.00001)	Antarctic coastal seawater
	*Legionella antarctica* (2708020)	55 (≤ 0.00001)	Antarctic lake
	*Nakamurella antarctica* sp. (1902245)	18 (≤ 0.00001)	Antarctica South Shetland Islands soil

Obs.: Taxonomic annotation of bacterial species with less than 10 reads were not included in the analysis. Stages 1: the beginning of the expedition; 2: middle of the expedition; 3: end of the expedition. *Information retrieved from National Centre for Biotechnology Information (NCBI) accession numbers where the closest related sequences were obtained through Blastx (search protein databases) search.

**Fig. 3:**
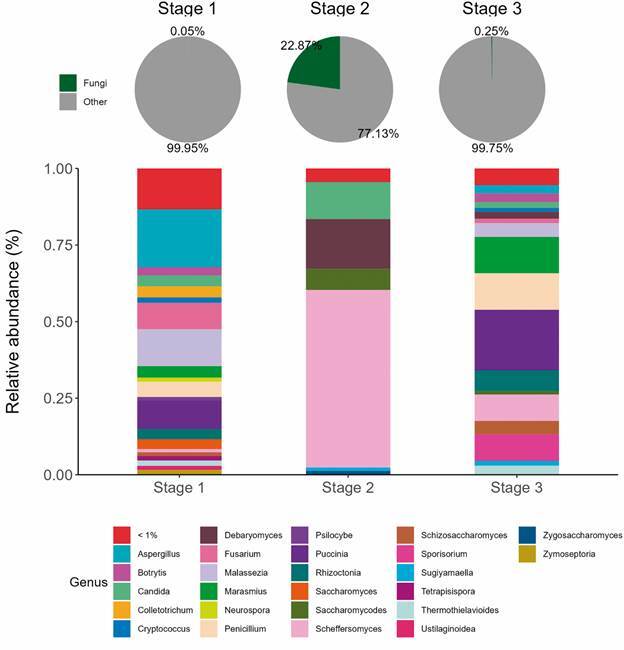
(A) Percentage (%) of fungi phyla (*Basidiomycota* and *Ascomycota*) obtained in each metagenomic library concerning total reads assigned. (B) Relative abundance of fungi genera obtained in each expedition stage of the Navy Polar ship (2019/2020).

Regarding the *Eukarya* domain and the *Fungi* Kingdom, two phyla were identified in all metagenomic libraries: *Basidiomycota* and *Ascomycota*. These two phyla corresponded to 0.05%, 22.8%, and 0.25% of the total reads obtained in stages 1, 2, and 3, respectively (Fig. 3A). *Basidiomycota* was identified in low abundance considering all reads assigned in stages 1, 2 and 3 (0.01%, 0.1% and 0.13%, respectively). *Ascomycota* represented 22.7% of all reads set in stage 2, with low abundance in stages 1 and 3 (0.03% and 0.11%, respectively).

*Aspergillus*, *Malassezia*, *Puccinia*, and *Fusarium* were the most abundant genus present in stage 1 (18.9%, 12%, 9.2%, and 8.6%, respectively), while other genera represented less than 5% within *Eukarya* domain (Fig. 3B). In stage 2, *Scheffersomyces* genus was predominant (57.9%), followed by *Debaryomyces* (16.2%), *Candida* (12%), *Saccharomycodes* (6.8%), and other less than 5% (Fig. 3B). *Puccinia*, *Penicillium*, *Marasmius*, *Sporisorium*, *Scheffersomyces* and *Rhizoctonia* were the most abundant genus present in stage 3 (19.7%, 11.9%, 11.8%, 8.6%, 8.6%, and 6.8%, respectively), while another genus were less abundant (≤ 5%) (Fig. 3B).

Some protozoans were observed in the pooled samples but in very low abundance (≤ 0.001%) considering all microbiome fractions. In stage 1 the genus *Babesia* was the most abundant and accounted for 1% of the reads assigned in the *Eukarya* domain, while other genera represented less than 0.05%. The *Toxoplasma* genus was the most abundant in stage 2, representing 0.04% of reads within the *Eukarya* domain, followed by *Babesia* (0.02%). In stage 3, the *Cryptosporidium* genus was predominant (0.6%) of the total reads belonging to the *Eukarya* domain, followed by *Babesia* (0.1%).

**Fig. 4:**
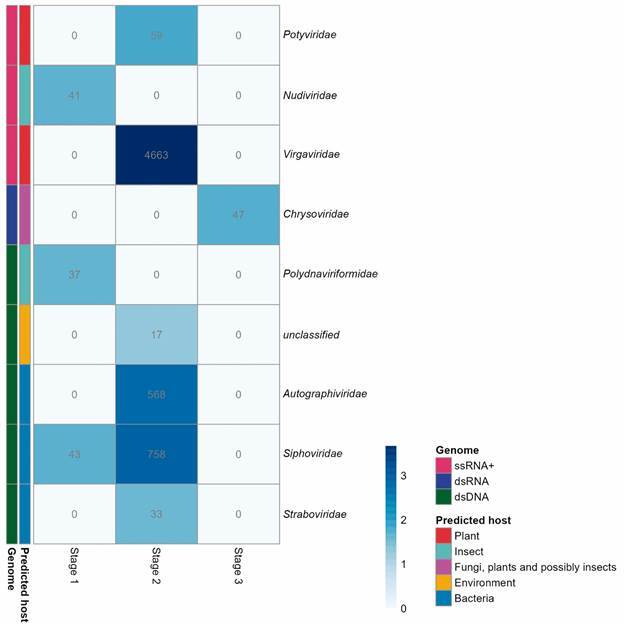
heatmap profile showing the relative abundance of viral families detected in each pooled sample of the ship’s indoor surfaces during different expedition stages to Antarctica (2019/2020). Each cell contains the number of reads that passed all the selection criteria. Data spanned from white (low relative abundance) to dark blue (high relative abundance), as illustrated by the color scale (log_10_).

*Virome* - Of the total reads classified in the virome, the highest abundance was observed in the pooled sample of stage 2 (97%), against 1.9% ranked in stage 1 and 1.1% in stage 3. Viruses with RNA genomes were the most abundant in the metagenomic libraries (77%), while viruses with DNA genomes corresponded to 23% of the total viral hits classified (Fig. 4). Fig. 4 shows the relative abundance of viral families obtained in each metagenomic library, their representative genomes, and predicted viral hosts. In total, eight viral families were identified in the virome. In stage 1, three viral families were identified: *Siphoviridae* [dsDNA] (35.5%), *Nudiviridae* [ssRNA+] (34%), and *Polydnaviriformidae* [dsDNA] [30.5%] (Fig. 4). In stage 2, a higher diversity of viral families was identified, being single-strand RNA viruses (ssRNA+) that infect plants (*Virgaviridae* family) predominant in this pooled sample (76.6% of the viral hits), followed by *Siphoviridae* [dsDNA] (12.4%), *Autographiviridae* [dsDNA] (9.5%), *Potyviridae* [ssRNA+] (1%), and *Straboviridae* [dsDNA] (0.5%) (Fig. 4). In stage 3 prevailed viruses of the *Chrysoviridae* [dsRNA] family (100%) (Fig. 4).

Reads were reassembled to refine viral analysis using metaSPAdes (Table IV). Table IV shows the description, distribution by length and similarity search using Blastx of the assembled reads per sample. A higher number of contigs was observed in stage 2 compared to stages 1 and 3 (Table IV). In total, 38 viral contigs were assembled.

**TABLE IV:** *De novo* assembly: total number of contigs, distribution by length and similarity search using Blastx for viral contigs in each pooled sample from indoor surfaces of a Navy Polar ship during the Antarctic expedition 2019/2020

	Assembly (metaSpades)						Similarity search (Blastx)	
Sample	number of contigs	length of the largest contig	number of contigs (> = 1000 bp)	number of contigs (> = 150 bp)	N50*	L50**	contigs (> = 150 bp) with hits against RefSeq	Viral contigs (> = 150 bp)
1	22,655	1,434	5	11,585	242	6,263	2,307	2
2	52,658	2,372	24	31,380	272	14,855	8,234	25
3	7,293	2,730	16	4,451	273	2,020	2,648	11

*N50 - length such that sequence contigs of this length or longer include half the bases of the assembly; **L50 - number of sequences contigs that are longer than, or equal to, the N50 length and therefore include half the bases of the assembly.

In stage 1, Propionibacterium phage PHL041M10 of the Pahexavirus genus and Actinomyces virus Av1 belonging to genera *Dybvigvirus* were identified (Table V). A greater richness of viral species was identified in samples from stage 2, collected four months after the beginning of the expedition (Table V). Single-strand RNA viruses (ssRNA+) that infect plants (represented by the Tobacco mosaic virus from the *Virgaviridae* family) were predominant in this pooled sample (Table V). Watermelon mosaic virus (*Potyviridae* family, *Potyvirus* genus, ssRNA+) was also identified, besides other plant (vegetal/fruit)-infecting viruses, such as Tomato brown rugose fruit virus and Pepper Mild Mottle Virus (PMMoV) (Table V). We also identified some species of the *Pahexavirus* genus, which are dsDNA bacteriophages with the predicted hosts (*Propionibacterium* sp., and *Propionibacterium acnes*). Data on S*taphylococcus*-infecting phages in stage 2 reflected the presence of *Staphylococcus* sp. in this pooled sample, corroborating microbiome findings. Another member of the *Siphoviridae* family (Psychrobacter phage Psymv2) was identified, containing sequences closely related to an open reading frame (ORF) coding a phage head-tail connector (RefSeq YP_009017594.1) (Table V). *Flavobacterium* infecting phages and viruses associated with phytopathogens of potato (Dickeya phage vB_DsoP_JA10 and Dickeya phage Ninurta) were observed, beyond enterobacteria infecting phages (Yersinia phage phiR1-37) and Acinetobacter virus Acj61 (Table V) infecting the predicted host *Acinetobacter johnsonii* found in aquatic sources, human skin, and animals.

**TABLE V:** Viral species (contigs) identified in the pooled samples from different sampling periods (1, 2 and 3) in indoor surfaces from the Brazilian Polar ship during the XXXVIII Brazilian Antarctic expedition, 2019/2020

Library	Virus taxonomy			Top Blastx hit				
	Order	Family	Genus	Max score	E-value	Identity (%)	Accession number	Protein
1								
NODE 855 - Propionibacterium phage PHL041M10	*Caudovirales*	*Siphoviridae*	*Pahexavirus*	114	1.28e-30	90.625	YP_009152679.1	hypothetical protein ACQ82_gp18
NODE 2838 - Actinomyces_virus_Av1	*Caudovirales*	*Podoviridae*	*Dybvigvirus*	84	1.65e-18	81.250	YP_001333657.1	hypothetical protein AV1_gp04
2								
NODE 4 - Tobacco mosaic virus	*Martellivirales*	*Virgaviridae*	*Tobamovirus*	1180	0.0	99.345	NP_597746.1	Replicase
NODE 35 - Tobacco mosaic virus	*Martellivirales*	*Virgaviridae*	*Tobamovirus*	519	0.0	98.438	NP_597748.1	Movement protein
NODE 170 - Tobacco mosaic virus	Martellivirales	Virgaviridae	*Tobamovirus*	409	3.30e-140	99.492	NP_597747.1	RNA polymerase
NODE 503 - Tobacco mosaic virus	*Martellivirales*	*Virgaviridae*	*Tobamovirus*	291	3.24e-94	100.0	NP_597747.1	RNA polymerase
NODE 526 - Sewage-associated gemycircularvirus 4	*Geplafuvirales*	*Genomoviridae*	*Gemykrogvirus*	174	4.10e-50	74.528	YP_009115508.1	replication-associated protein
NODE 1618 - Tobacco mosaic virus	*Martellivirales*	*Virgaviridae*	*Tobamovirus*	296	6.18e-91	97.857	NP_056764.1	Replicase
NODE 1632 - Propionibacterium phage PHL141N00	*Caudovirales*	*Siphoviridae*	*Pahexavirus*	226	2.70e-73	97.656	YP_009152612.1	hypothetical protein ACQ69_gp22
NODE 2080 - Tomato brown rugose fruit virus	*Martellivirales*	*Virgaviridae*	*Tobamovirus*	254	8.81e-76	99.160	YP_009182169.1	126 kDa replicase
NODE 3191 - Watermelon mosaic virus	*Patatavirales*	*Potyviridae*	*Potyvirus*	265	4.77e-84	96.094	YP_077276.1	nuclear inclusion b
NODE 4361 - Psychrobacter phage Psymv2	*Caudovirales*	*Siphoviridae*	unclassified	173	4.29e-53	86.735	YP_009017594.1	phage head-tail connector
NODE 5086 - Watermelon mosaic virus	*Patatavirales*	*Potyviridae*	*Potyvirus*	205	3.77e-60	96.875	YP_077271.1	cylindrical inclusion protein
NODE 5467 - Flavobacterium_phage_vB_FspM_immuto_2-6A	Caudovirales	*Myoviridae*	unclassified	164	4.20e-45	70.588	YP_010114609.1	capsid assembly protein
NODE 6257 - Flavobacterium_phage_vB_FspM_immuto_2-6A	*Caudovirales*	*Myoviridae*	unclassified	171	1.92e-51	81.250	YP_010114449.1	hypothetical protein KNV73_gp234
NODE 9505 - Yersinia_phage_phiR1-37	*Caudovirales*	*Myoviridae*	unclassified	150	1.15e-39	73.585	YP_004934333.1	putative RNA-polymerase beta-subunit
NODE 10205 - Acinetobacter_virus_Acj61	*Caudovirales*	*Myoviridae*	*Lasallevirus*	102	1.78e-26	79.688	YP_004009647.1	hypothetical protein Acj61p030
NODE 12095 - Staphylococcus_virus_BS1	*Caudovirales*	*Herelleviridae*	*Baoshanvirus*	107	8.63e-25	89.091	YP_009799535.1	hypothetical protein HOS99_gp031
NODE_12637 - Watermelon mosaic virus	*Patatavirales*	*Potyviridae*	*Potyvirus*	189	2.89e-56	95.652	YP_077269.1	helper component-protease
NODE_12735 - Watermelon mosaic virus	*Patatavirales*	*Potyviridae*	*Potyvirus*	191	1.41e-55	98.925	YP_077271.1	cylindrical inclusion protein
NODE_14474 -Watermelon mosaic virus	*Patatavirales*	*Potyviridae*	*Potyvirus*	134	8.70e-38	98.718	YP_077274.1	viral protein genome-linked
NODE_14674 - Staphylococcus phage SA11	*Caudovirales*	*Herelleviridae*	*Silviavirus*	81.6	5.46e-18	72.0	YP_007005609.1	hypothetical protein F422_gp134
NODE_15557 -Tobacco mosaic virus	*Martellivirales*	*Virgaviridae*	*Tobamovirus*	96.154	2.94e-43	159	NP_056764.1	Replicase
NODE_17934 - Dickeya phage vB_DsoP_JA10	*Caudovirales*	*Autographiviridae*	*Ningirsuvirus*	85.1	5.18e-19	100.0	YP_009811103.1	putative internal core protein
NODE_18273 - Watermelon mosaic virus	Patatavirales	Potyviridae	*Potyvirus*	163	1.15e-44	91.667	YP_077181.1	polyprotein
NODE_18902 - Pepper mild mottle virus	*Martellivirales*	*Virgaviridae*	*Tobamovirus*	165	3.42e-49	97.590	NP_619742.1	movement protein
NODE_25014 - Dickeya phage Ninurta	*Caudovirales*	*Autographiviridae*	*Ningirsuvirus*	142	6.65e-41	93.243	YP_009801146.1	tail tubular protein B
3								
NODE_51 - Penicillium chrysogenum virus	*Ghabrivirales*	*Chrysoviridae*	*Alphachrysovirus*	400	2.20e-129	90.777	YP_392482.1	RNA-dependent RNA polymerase
NODE_213 - Penicillium chrysogenum virus	*Ghabrivirales*	*Chrysoviridae*	*Alphachrysovirus*	323	4.49e-102	99.355	YP_392483.1	major capsid protein
NODE_635 - Penicillium chrysogenum virus	*Ghabrivirales*	*Chrysoviridae*	*Alphachrysovirus*	195	1.89e-55	97.872	YP_392485.1	hypothetical protein
NODE_636 - Penicillium chrysogenum virus	*Ghabrivirales*	*Chrysoviridae*	*Alphachrysovirus*	274	9.50e-85	100.0	YP_392485.1	hypothetical protein
NODE_961 - Penicillium chrysogenum virus	*Ghabrivirales*	*Chrysoviridae*	*Alphachrysovirus*	113	1.24e-26	98.529	YP_392483.1	major capsid protein
NODE_1206 - Dragonfly larvae associated circular virus-2	unclassified	unclassified	unclassified	148	4.90e-42	78.889	YP_009001739.1	replication-associated protein
NODE_1660 - Penicillium chrysogenum virus	*Ghabrivirales*	*Chrysoviridae*	*Alphachrysovirus*	199	2.56e-57	98.947	YP_392485.1	hypothetical protein
NODE_2251 - Pepper mild mottle virus	*Martellivirales*	*Virgaviridae*	*Tobamovirus*	103	4.66e-26	100.0	NP_619743.1	coat protein
NODE_2389 - Penicillium chrysogenum virus	Ghabrivirales	*Chrysoviridae*	*Alphachrysovirus*	145	1.82e-38	100.0	YP_392482.1	RNA-dependent RNA polymerase
NODE_2885 - Botryotinia fuckeliana partitivirus 1	*Durnavirales*	*Partitiviridae*	unclassified	115	4.75e-28	72.727	YP_001686789.1	RNA-dependent RNA polymerase
NODE_4083 - Penicillium chrysogenum virus	*Ghabrivirales*	*Chrysoviridae*	*Alphachrysovirus*	149	4.98e-40	100.0	YP_392482.1	RNA-dependent RNA polymerase

*Contigs identified based on best score and lower e-value through Blastx (search protein database).

At the end of the expedition, a predominance of fungi-infecting viruses (dsRNA) of the genus *Alphachrysovirus* (*Chrysoviridae* family) was observed, as well as a member of the *Partitiviridae* family and PMMoV (Table V).

## DISCUSSION

*Metagenomic data* - In this study, we aimed to evaluate the microbial diversity present on indoor surfaces of a polar ship in different stages of an expedition from Brazil to the Antarctic continent, using a shotgun metagenomic approach. The main results demonstrated that the most abundant bacterial phyla corresponding to the total fraction of microbiome were *Proteobacteria*, followed by *Firmicutes*, *Actinobacteria*, and *Bacteroidetes* and according to other studies conducted to evaluate the microbiome in indoor surfaces of built environments or transportation facilities.^24^ ^,^ ^36^ ^,^ ^37^ However, it was possible to observe variations in the relative abundance of microbial families and genera during different expedition stages. At the beginning and end of the expedition, with a limited number of passengers on the ship, it was possible to observe the prevalence of bacterial genera commonly present in the environment, such as *Pseudomonas* spp. (stage 1) and *Massilia* spp. (stage 3). Pseudomonas is a gram-negative, ubiquitous bacteria (widely found in diverse environments) of *Pseudomonadaceae*. Genera *Massilia* sp. are psychrophilic or mesophilic and are generally considered environmental organisms rather than animal-associated and have already been isolated from different settings, such as freshwater, glaciers, rocks, and air samples.^38^

A different profile of bacterial genera was observed in stage 2 (during the expedition). *Cutibacterium* was the most relatively abundant microbial genus in stage 2, and similar findings were observed by Danko et al.,^24^ which identified a higher abundance of *Cutibacterium acnes* (known human skin commensal) in indoor surfaces of urban transportation facilities. *Staphylococcus* was the second bacterial genus most prevalent in this stage and comprises gram-positive bacteria that can be found both on the human skin and on the nasal mucous. The abundance of bacterial genus commonly present in indoor surfaces of built environments or transportation facilities with human presence has been documented.^23^ ^,^ ^24^ ^,^ ^37^ The top taxa associated with indoor environments were recognisable as microbes associated with humans (*e.g.*, *Corynebacterium*, *Streptococcus*, *Enterobacteriaceae*, *Staphylococcus*, *Propionibacterium*, *Lactococcus*)^23^ and are also comparable with our results, corroborating the findings of our study for pooled samples of the middle of the expedition, when passengers were confined. This distinct pattern may be related to the more significant number and circulation of people and activities on the ship during the middle of the expedition since the nature of human contact and human behaviour highly influences indoor surfaces.^6^ ^,^ ^8^ ^,^ ^23^

It is worth noting that samples from stage 2 (collected during the expedition) were stored at 4ºC before metagenomic analysis, and this could favor the growth of some bacterial groups, specifically of the *Gammaproteobacteria* class.^39^ However, this is a controversial discussion since other studies confirmed that the phylogenetic structure and diversity of communities were not significantly influenced by storage temperature or duration of storage.^39^ ^,^ ^40^ Lauber et al.^40^ have demonstrated that the relative abundances of most taxa were largely unaffected by temperature even after 14 days of storage in a saline buffer in a study on assessing bacterial community structure in soil and human-skin-associated samples. Moreover, the bacterial relative abundance data in these samples are consistent and comparable to other metagenomic and microbiome studies in indoor environments and transportation facilities with a high circulation of people.^24^ ^,^ ^36^

In the middle of the expedition (stage 2), it was also possible to observe a greater abundance of species in the Eukarya domain, especially fungi of the *Ascomycota* phyla. The expected higher humidity at this stage of the voyage and different activities on the vessel, including collecting water samples, crew embarking and disembarking on the sea, food cooking, and use of restrooms, among others, might allow for a more significant proliferation of fungi. Unfortunately, measuring the humidity inside the ship during the collection periods was impossible. Still, the average temperature inside the ship during the voyage ranges from 20 to 25ºC, which can also be an appropriate temperature for the growth of many species of fungi. Genera of yeasts (*Scheffersomyces* and *Debaryomyces*) were the most abundant in this stage, followed by *Candida*. Ascomycetous yeasts are widely distributed in nature, and most are saprotrophs and represent important decomposers, but some species are pathogens of plants and animals as well. Yeasts are generally considered to be mesophiles, and optimal growth temperatures are around 25ºC. *Candida* represents a fungi genus that colonises the human organism without causing infections. Some are opportunistic pathogens, like *Candida albicans*, for example.

The abundance of fungi in stages 1 and 3 was shallow concerning stage 2. The presence of fungi at the beginning and end of the expedition could also be related to the temperature range (average temperature in Rio de Janeiro in October 2019 and April 2020 varied between 20 to 25ºC and 25 to 30ºC, respectively) (INMET - Instituto Nacional de Meteorologia - https://clima.inmet.gov.br/progt), thus favouring the proliferation of other fungi species found mainly on surfaces, including molds in stored food or residues and wood, among other surfaces. We collected samples from the air conditioning system, which could also be an essential source of fungi. These microorganisms are usually filtered from the ventilation system to prevent particles and microbes from entering the indoor air.^41^ ^,^ ^42^ However, *Cladosporium*, *Penicillium*, and *Aspergillus* have been detected on passenger ships.^42^

Unfortunately, a limitation of the study is that we did not perform assays with individual samples collected in the kitchen, bathroom, bedroom, and laboratory areas. Therefore, we could not accurately observe the microbiome characteristics of each surface and compartment.

However, the techniques allowed us to observe some bacterial species related to the Antarctic continent during the different sampling stages. These bacterial species observed are part of the environmental microbiome, not being characterised as potential animal or human pathogens. In addition, the abundance of these species was very low considering the microbiome’s total fraction. Studies on the viability of these microorganisms would be necessary to assess whether there is any risk of dispersion to other continents, adaptation, and future ecological impact scenarios.

*Virome* - Most of the studies performed to evaluate the microbiome of internal surfaces of transportation facilities are directed towards analysing the taxonomic composition of bacteria using 16S rRNA gene sequencing.^6^ ^,^ ^23^ The shotgun metagenome can explore all available DNA in a sample without a specific target. In our study, the methodology used was also directed to capture DNA and RNA, focusing mainly on detecting viruses with RNA genome. To date, few studies on the microbiome characterisation on indoor surfaces in transportation facilities or built environments have focused on the analysis of RNA viruses, and much knowledge about their distribution and patterns of occurrence in the environment is still needed.^6^ ^,^ ^7^ ^,^ ^8^ ^,^ ^23^ ^,^ ^24^

Virome analysis demonstrated that RNA viruses prevailed over DNA viruses in the metagenomic libraries. In stage 1 it was possible to identify *Pahexavirus* that infect bacteria of the genus *Propionibacterium* sp., which are pleomorphic, occasionally branching bacilli that are the normal flora of the skin, conjunctiva, external ear canal, and exposed mucous membranes.^43^ Single-strand ssRNA+ viruses belonging to the *Nudiviridae* and *Polydnaviriformidae* families recognised to infect insects were also detected in this stage. One contig in this sample was assigned to NODE 2838 - Actinomyces_virus_Av1 (*Podoviridae* family) commonly found in humans’ mouths. These findings indicate that insect-infecting viruses and commensal microorganisms of the human skin or mucous membranes prevailed on the sampled surfaces without viral pathogens that cause human diseases. The low number of people circulating inside the ship during this sampling period and the previous cleaning of the vessel before receiving the crew and researchers to initiate the expedition could explain the results.

A higher abundance of viral hits and a larger variety of assigned species was observed in pool 2 (swab samples collected in the vessel during the expedition). The result is consistent with many co-livings on the ship (military crews and researchers). In this context, even with the routine cleaning and hygienic measures of the ship´s compartments and surfaces, the large circulation of people could explain a greater spread of viruses in these environments. No known human pathogenic viruses were identified in this stage of the trip. The absence of such viruses was corroborated by the lack of any report of disease outbreaks, such as gastroenteric or respiratory diseases, frequently reported in such confined settings.^2^ ^,^ ^5^ ^,^ ^7^ ^,^ ^9^

A diversity of bacteriophages within the *Siphoviridae* family infecting commensal skin bacteria was detected, including *Staphylococcus* sp., which is genera of Gram-positive bacteria and is part of the normal microbiota of the mouth, skin, intestine, or upper respiratory tract. Bacteriophages, including *Staphylococcus* and *Propionibacterium* phages, were also observed in a similar study using shotgun metagenomic analysis to characterise the microbiome of an enclosed public transport (aircraft), where a low abundance of human viral pathogens was also reported.^26^

Members of the *Virgaviridae* family were dominant in pool 2, mainly represented by Tobacco mosaic virus (TMV) species. TMV has an extensive host range, and it is known to infect members of several plant species, including tobacco, tomato, pepper (*Solanaceae* family), cucumbers, and ornamental flowers, among others. Other viruses infecting plants, vegetables, or other foods, such as Pepper Mild Mottle Virus (PMMoV), Tomato Brown Rugose Fruit Virus, and Watermelon Mosaic Virus, were also detected during this expedition stage.

Among these detected viruses, PMMoV (*Virgaviridae* family) is abundant in the human gut, consequently in feces and raw sewage and is considered an essential human fecal viral marker in aquatic environments.^44^ ^,^ ^45^ ^,^ ^46^ However, we cannot confirm the location of contamination caused by PMMoV due to the pooling of samples. Similarly, this voyage stage also detected a Gemykrogvirus (Contig 526 - Sewage-associated gemycircularvirus 4). The presence of plant-infecting viruses, such as PMMoV, was expected, particularly considering that several sampling sites were close to the crew’s dining and kitchen areas. Moreover, due to the high ingestion during regular diet and faecal excretion of PMMoV, studies have considered this virus as an excellent viral marker of human faecal contamination in the environment.^45^ ^,^ ^46^

Initially, to screen for human faecal contamination using viral markers, we screened the 91 individual swab samples collected throughout the expedition for human adenovirus (HAdV) using a qPCR protocol.^47^ HAdVs are important viral markers for assessing human faecal environmental contamination.^45^ None of the samples tested positive for HAdV. Similarly, by the metagenomic approach, we have yet to identify HAdVs.

In the indoor surface samples, it was possible to identify only one viral species that is probably native to the Antarctic continent (Psychrobacter phage Psymv2) (NCBI RefSeq YP_009017594.1). This bacteriophage was initially isolated from a bacterium of the genus Psychrobacter (*Psychrobacter* sp. MV2) identified in soil samples in Miers Valley, in the McMurdo Dry Valleys, South Victoria Land, Antarctica.^48^ Subsequently, Psychrobacter phage Psymv2 was found to be the most abundant viral species observed in surface and bottom sea samples from Prydz Bay viromes (Antarctica) in 2015.^49^ The host bacteria strains of this phage include some members of the genus *Psychrobacter*, which have been isolated from a wide range of habitats, including surface and deep-sea waters, deep-sea sediments and soil, especially from the Antarctic region, and are also widespread in cold Antarctic environments.^48^ ^,^ ^49^ At the end of the expedition, they were not identified.

In stage 3, occurred the predominance of viruses with a dsRNA genome that infects fungal species of the *Penicillium* genus. *Penicillium* was the second genus of fungi most abundant among eukaryotes in this stage of the trip, according to microbiome analysis.

This study documents a first screening to assess the virome at different stages of an expedition to Antarctica, tracking viral communities in a closed environment. Swab-based methods have been used to explore viral contamination on different types of surfaces using RT-qPCR.^50^ In contrast, the sensitive next-generation sequencing (NGS) technique and shotgun metagenomic to detect specific pathogenic viruses in surface samples or indoor environments still need to be thoroughly evaluated.^24^ ^,^ ^26^ In a study aimed to investigate the microbiome in urban transit systems, including subways and buses in several cities around the world, researchers have not reported archaea or viruses in such samples.^24^ Some limitations for the absence of viral detection in these transport facilities were attributed to the DNA extraction methods used, limitations in sequencing depth, or missing annotations in reference databases used for classification, highlighting the challenges for obtaining the virome in these environments.^24^ Moreover, new sampling devices have been tested for microbial sampling, including upgraded swabs (different materials), polyester wipes, macrofoam sponges, adhesive tapes, biological sampling kit (BiSKit; macrofoam), witness coupons, dust, and bulk sampling which could be more effective in concentrating and detecting the microbial population found in indoor surfaces.^51^

In our study, frequently cleaning the navy vessel may reduce the presence of pathogenic viruses. Nevertheless, it is essential to emphasise that the absence of viral enrichment protocols can reduce the scope of viral assessment to only the most abundant viruses in the samples. Therefore, viral enrichment protocols can help observe viruses present in lesser abundance in the samples,^52^ but it limits the microbiome study by excluding other types of microorganisms. Target-specific detection techniques could be used as a supplementary approach in pathogen surveillance to analyse known pathogens. This approach could be beneficial, especially in the context of pandemic situations.

Our analysis started in October 2019, before the COVID-19 pandemic. Therefore, future virome analyses coupled with the search for specific pathogenic viruses could be an exciting strategy to track viral diversity, especially for viruses of public health importance.

*Final considerations* - Although we did not identify a high abundance of pathogenic microorganisms, it is essential to emphasise that their detection through (meta)genomic analysis does not indicate a direct risk to humans or the environment. Further work is necessary to assess viability and risk of infection. The health risk is associated with several factors, including stability on fomites, the number of infectious agents contacted by the fingerpad, the efficiency of self-inoculation (*i.e.*, transfer of the pathogen from fingerpad to the mouth, nasal cavity, eyes, or other bodily location where infection may occur), the infectious dose of the organism and the individual’s susceptibility.^1^ ^,^ ^16^ Therefore, all these variables should be considered in a health risk analysis.

Cleaning and disinfection of contaminated surfaces are frequently implemented measures to control the transmission of pathogens in indoor environments and reduce human fingerprints.^53^ ^,^ ^54^ Still, microorganisms that are sporulated or that form cysts, like some protozoans species, may be more resistant to disinfection. The risk of infection can also be reduced by increasing ventilation in some locations when possible.^11^ The use of high-efficiency particulate air filters and ultraviolet germicidal treatment in the ventilation system are significant effective measures.^11^ ^,^ ^42^ In addition, implementing a comprehensive outbreak prevention and control strategy could reduce the impact of viral infection on vessels, particularly relevant in pandemic situations.^13^

Despite some limitations, this study demonstrates that shotgun metagenomics may be appropriate to describe the microbial diversity of indoor surfaces of transportation facilities, such as cruise or navy ships. Moreover, results suggest that microbial communities on ship’s indoor surfaces contain a metapopulation of human skin/mucous commensals and environmental generalists, with variations corresponding to the number of passengers, activities on board, and environmental exposures. This study also evaluated and tracked the abundance of autochthonous or allochthones environmental species between continents and crew, particularly relevant in the Antarctic context.

*Data availability* - The data that support the findings of this study are openly available in [Sequence Read Archive (SRA) (NCBI)] at [https://www.ncbi.nlm.nih.gov/sra], reference number [PRJNA850925].
